# Urinary and Plasma miRNAs in the Early Detection of Acute Kidney Injury and Their Possible Role as Therapeutic Targets

**DOI:** 10.3390/jcm14072306

**Published:** 2025-03-28

**Authors:** Anna Clementi, Grazia Maria Virzì, Claudio Ronco, Paola Monciino, Monica Zanella

**Affiliations:** 1 Department of Nephrology and Dialysis, Santa Marta and Santa Venera Hospital, 95024 Acireale, Italy; a.clementi81@virgilio.it (A.C.); paola.monciino@aspct.it (P.M.); 2 Department of Nephrology, Dialysis and Transplant, St Bortolo Hospital, 36100 Vicenza, Italy; monica.zanella@aulss8.veneto.it; 3 IRRIV—International Renal Resarch Institute Vicenza, 36100 Vicenza, Italy; cronco@goldnet.it

**Keywords:** acute kidney injury, micro-RNA, miRNAs, non-coding RNA, ncRNA, extracellular vesicles

## Abstract

Acute Kidney Injury (AKI) is a severe clinical condition featured by a rapid decrease in kidney function in a short period of time. AKI, which is often secondary to sepsis, ischemia-reperfusion and drug toxicity, is associated to high morbidity and mortality. Moreover, it contributes to the development of chronic kidney disease (CKD), due to maladaptive or incomplete repair mechanisms, resulting in renal fibrosis. Small non-coding RNA has recently emerged as a novel biomarker for the early detection and treatment of AKI. In particular, microRNAs (miRNAs) are non-coding RNA molecules of 21–25 nucleotides regulating the expression of protein-coding genes through sequence-specific recognition. Due to their high stability in biological fluids, such as urine and plasma, they can be reliably analyzed and quantified, and for this reason they can be considered potential diagnostic and therapeutic biomarkers. Specifically, miRNAs have been demonstrated to predict AKI before the increase in creatinine levels, thus improving the management of this syndrome. In this review, we provide a comprehensive overview of the role of urinary and plasma miRNAs in the early detection and treatment of AKI.

## 1. Introduction

Acute Kidney Injury (AKI) is a severe clinical syndrome characterized by a rapid decrease in kidney function in a short period of time, often resulting in water imbalance and electrolytes alterations, such as hyperkalemia, hyponatremia, and metabolic acidosis. If they are not promptly corrected, they can result in serious complications, including cardiac arrhythmias, pulmonary edema, and even death. Sepsis, ischemia-reperfusion, drug toxicity, urinary tract obstruction, and rhabdomyolysis are among the most frequent and critical causes of AKI. All these conditions lead to renal dysfunction through distinct mechanisms, but they all result in a rapid decline in kidney function, posing significant risks to patient health. Understanding how each factor may contribute to the development of AKI is essential for an early diagnosis, a better management, and the prevention of the possible progression of renal damage [1].

While the prevalence of AKI has been reported to be 23.2% in hospitalized patients [2], in intensive care unit its prevalence increases to 57.3% [3]. The mortality of AKI ranges from 3.4% in stage 1 to 24.1% in patients undergoing dialysis [3]. In particular, a single episode of AKI is associated with a significant mortality risk, with an episode of stage 1 AKI complicating a critical illness being independently associated with an increase in 10-year mortality [4].

Moreover, this clinical syndrome often has a poor long-term outcome, due to the possible development of chronic kidney disease (CKD), and in some cases irreversible end-stage renal disease (ESRD). The transition from AKI to CKD can occur through different mechanisms, including inflammation and fibrosis, which both impair the ability of the kidney to recover [5]. In fact, surviving renal tubular cells may not always be able to replace the damaged tissues, resulting in renal fibrosis and prolonged kidney dysfunction [5]. In specific clinical settings, AKI can lead directly to irreversible ESRD. This is particularly common in patients who experience recurrent episodes of AKI, in those with subclinical pre-existing kidney disease, or in those who are exposed to nephrotoxic agents or other risk factors [5].

Therefore, the early detection of AKI is absolutely fundamental not only to anticipate the management of this clinical condition but also to prevent its potential complications and long-term consequences. Indeed, the early diagnosis of AKI allows a rapid beginning of the therapy with a higher probability of complete recovery. Unfortunately, despite significant advancements in the understanding of the pathogenesis of AKI and its underlying mechanisms, there is still no gold standard for the early diagnosis of this condition [6,7].

A lot of literature has been produced on the role of novel biomarkers over the past 10 years [8], reflecting the growing need for more reliable and earlier diagnostic tools. These novel biomarkers are being explored with the goal of improving the detection, management and prognosis of AKI, particularly in its early stages when traditional markers such as serum creatinine (Scr) and urine output may not be sufficiently sensitive [9]. They include neutrophil gelatinase-associated lipocalin (NGAL), the cysteine protease inhibitor Cystatin C (Cys-C), kidney injury molecule 1 (KIM-1), the product of insulin like growth factor binding protein 7 (IGFBP-7) and tissue inhibitor of metalloproteinase 2 (TIMP2). With areas under the curve (AUC) of 0.67 for NGAL [10], 0.71 for Cys-C [11], 0.65 for KIM-1 [10], and 0.80 for the product of IGFBP7 and TIMP-2 [12], these biomarkers have shown a better performance in the diagnosis of AKI compared to creatinine and urea. Nevertheless, to date most of these markers have not been established in clinical practice. In the meanwhile, a large number of animal and human studies on the potential role of non-coding RNA (nc-RNA), and more specifically micro-RNA (miRNAs) in the diagnosis and therapy of AKI have been recently produced. In particular, miRNAs play a pivotal role in different biological functions, like cell differentiation, growth and proliferation, apoptosis, immune response and inflammation. Moreover, thanks to their effects on gene regulation, they are able to influence critical pathways involved both in kidney function and pathology. Therefore, they may help clinicians in the early diagnosis of acute kidney injury, as well as in the differentiation of AKI etiologies, such as sepsis, ischemic injury, drug and contrast toxicity. In this context, they represent promising biomarkers for AKI compared to traditional markers, and potential therapeutical targets.

### Aim of the Work

This review aims to describe the current knowledge about the role of micro-RNAs in the early detection of AKI and in its treatment, in order to improve the management of this syndrome, thus preventing the possible development of CKD and reducing mortality. This review is focused on the possible role of miRNA as both early diagnostic markers and potential therapeutic targets in the setting of AKI.

## 2. Material and Methods

A complete search strategy was carried out with the use of search strings across PubMed and Cochrane databases. The aim of this search strategy was a systematic revision of relevant studies and clinical trials regarding acute kidney injury, its early detection, and the possible role of biomarkers in its diagnosis and treatment. We reported the search strings used: (acute kidney injury OR (acute kidney damage)) AND (microRNAs OR miRNAs), (acute kidney injury OR (acute kidney damage)) AND (non-coding RNAs). Furthermore, PubMed was used to identify published papers with specific terms to elaborate and add details to our results. The references of the retrieved papers were used to find more literature. We restricted our bibliography research to the last 10 years. By targeting recent literature, we ensured that the references included were representative of the most current findings, reflecting the advances in diagnostic technologies, biomarker discoveries, and therapeutic interventions in the setting of acute kidney injury.

## 3. MiRNAs

Considerable research has recently reported that the non-coding RNAs (ncRNAs), especially micro RNAs (miRNAs), are involved in the pathophysiology of acute kidney injury. These small RNA molecules, once considered “junk” RNA, have now been recognized as essential regulators of gene expression, influencing a variety of cellular processes, such as inflammation, oxidative stress, tubular injury, fibrosis, and repair mechanisms in the renal environment.

In 2001, the completion of the human genome sequencing marked the beginning of extensive work aiming at characterizing the function of human genes. Even though initially the human genome was thought to be mostly composed of protein-coding genes, the revelation that only a small proportion of human genes was indeed able to encode proteins (2%) represented an important breakthrough in the characterization of the human genome. However, 80% of our genes are transcribed leading to the synthesis of RNA molecules. These findings by the ENCODE Project Consortium 16 implied that the majority of human genes are transcribed into non–protein-coding RNAs or more simply ncRNAs. This revelation has expanded the definition of the genome’s functional components, thus providing a new perspective on gene expression. Previously, it was widely believed that the primary function of genes was to encode proteins, which are responsible for most biological functions. However, the ENCODE project highlighted the immense complexity of the genome by showing that a large proportion of transcribed RNA does not code for proteins but instead produces ncRNAs.

The ncRNA family is a large and diverse class of RNAs with multiple functions [13]. According to a general consensus, the criteria defining a non-coding RNA as long or short depends on the number of nucleotides which is set to 200 by default. The small ncRNA consists of small nuclear RNA (sn-RNA), small nucleolar RNA (sno-RNA), repeat associated small interfering RNA (rasi-RNA), small cell osteosarcoma RNA (sco-RNA), micro RNA (miRNA), small interfering RNA (si-RNA), piwi-interacting RNA (piRNA) and transcription initiation RNA (ti-RNA) [13]. The micro RNAs are usually less than 25 nucleotides.

**Figure 1:**
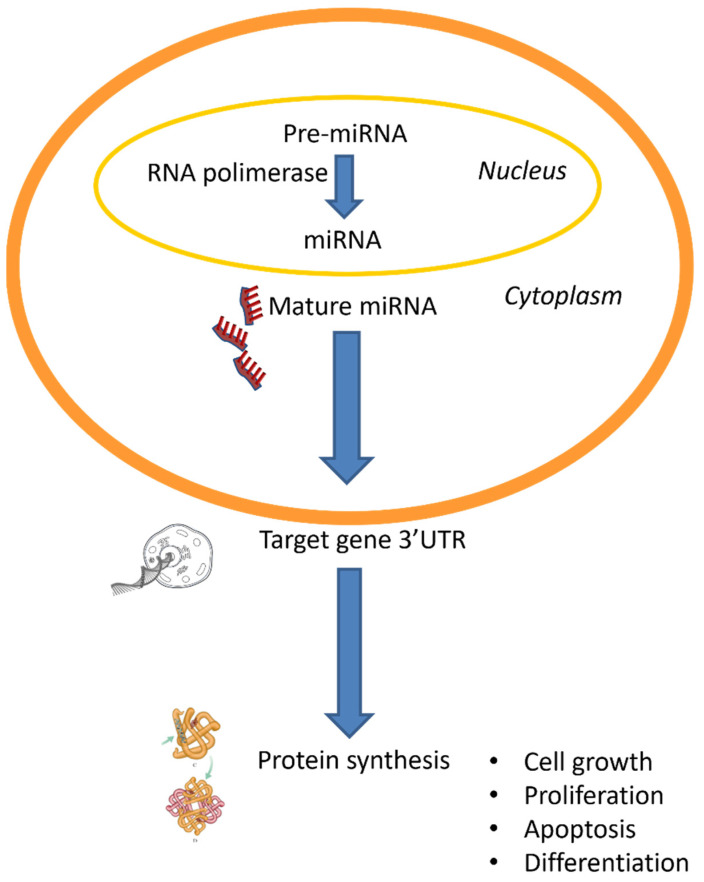
Release of miRNA and mechanisms involved in AKI pathogenesis.

MiRNAs are non-coding RNA molecules of 21–25 nucleotides regulating the expression of protein-coding genes through a sequence-specific recognition, binding to 3′- or 5′-untranslated region (3ʹUTR) of target messenger RNA (mRNA) or promoter sequences, thus modifying mRNA levels by post-transcriptional mechanisms [14,15] (Figure 1).

MiRNAs result from primary nuclear transcription through RNA polymerase, also referred to as long primary transcripts (pre-miRNAs), processed into 70- to 100- nucleotide sequences by RNase III Drosha. They are then exported into the cytoplasm where they are further processed to generate mature miRNA species of the form of double stranded RNAs [16]. MiRNAs have been shown to target a number of genes by either enhancing or inhibiting their expression via binding to the 3′-untranslated region (3′UTR) of their target genes. Within a given cell type, both cell-specific and diverse miRNAs can target several mRNAs. Moreover, plasma miRNAs are secreted from cells and enter the bloodstream to reach targeted cells, in a new communication approach in cell–cell or cell–organ signal transduction. In particular, the role of these molecules in specific cellular or physiological processes can be investigated through the deletion or inhibition of miRNA processing machinery [17].

As a result, miRNAs exert control on several vital processes of cellular activity and homeostasis including cell growth, proliferation, differentiation, and apoptosis [18,19]. Till date, approximately 3000 human miRNAs have been identified and they seem to regulate as many as 30% of all human mRNA transcripts [20]. This vast regulatory network indicates the profound influence of miRNAs on cellular processes, affecting virtually every aspect of cellular function and biological development. Because of their ability to regulate gene expression by binding to the 3′ untranslated regions (UTRs) of target mRNAs, miRNAs have a critical role in modulating protein synthesis, thus influencing a variety of biological pathways. Consequently, nearly every biological process is estimated to be under the control of miRNAs.

## 4. The Role of miRNAs in AKI

MiRNAs play a crucial role in kidney development, homeostasis and function, serving as key regulatory molecules responsible for the expression of genes involved in various renal processes [15]. Pavkovic et al. have identified 669 types of miRNA in normal human kidneys [21]. Moreover, it has been recently demonstrated that miRNAs are essential regulators of a number of signaling cascades involved in the pathogenesis of renal disease in vivo and in vitro. MiRNAs’ dysregulation is often responsible for the progression of kidney dysfunction. Additionally, the stability of miRNA in plasma and urine makes it a reliable indicator of pathological change [22]. Indeed, serum and plasma miRNAs, as well as urinary miRNAs have been investigated as potential biomarkers in the setting of hypertensive and diabetic nephropathy, nephrotic syndrome and IgA nephropathy, CKD and AKI [23]. Specifically, different miRNAs have been demonstrated to play an important role both in the diagnosis and in the therapeutical management of AKI (Table 1).

### 4.1. MiRNAs as Diagnostic Biomarkers and Therapeutic Targets

MiR-21-5p seems to be involved in the development of sepsis-induced AKI [24]. Indeed, it inhibits RUNX1 (runt-related transcription factor 1), responsible for increased serum inflammation, apoptosis and oxidative stress in the kidneys. The injection of miR-21-5p has been reported to be associated to an improvement of kidney function in a rat model of sepsis-induced AKI [24]. Even though further studies are necessary to better understand the exact mechanism through which miRNA-21-5p inhibits RUNX1, this miRNA may represent a potential therapeutical option for renal damage secondary to sepsis.

Also, MiR-374a-3p has been reported to play a role in the pathogenesis of sepsis-induced AKI through the interaction with small nuclear RNA host gene 5 (SNHG5), such that it is able to increase apoptosis levels and cytokine release [25]. In particular, SNHG 5 downregulation is associated with a reduced nuclear factor-ĸB (NF-ĸB) activity, induced by miR-374a-3p and toll-like receptor 4 (TLR4) interaction [26]. These data suggest that SNHG5 modulates sepsis-induced AKI through the axis miR374a-3p/TLR4/NF-ĸB which may represent a new pharmacological target in this clinical setting.

In the study of Huo et al, the predictive value for 28-day survival of miR-29-a and miR-10a-5p has been investigated in patients with AKI secondary to sepsis [26]. Correlations of miR-29a and miR-10a-5p with SCr, Cys-C, and KIM-1 were performed. Compared with the survivors, in the patients who died within 28 days significantly increased serum levels of Scr, Cys-C, KIM-1, miR-29a, and miR-10a-5p (*p* < 0.05) were found. Moreover, miR-29-a and miR-10a-5p were positively correlated with serum Scr, Cys-C, and KIM-1 levels, thus demonstrating to be good predictors for 28-day survival [26]. These molecules may represent novel predictor factors for mortality in septic AKI. Anyway, the standardization of miRNA quantification methods still represents a clinical challenge.

In the setting of contrast-induced AKI, which is characterized by acute nephropathy occurring after the infusion of intravascular contrast agents, there is no ideal biomarker for its early diagnosis. Gutierrez-Escolano et al. recently hypothesized that specific circulating miRNA might serve such a role [27]. In the kidney tissue of rats used as a model of contrast-induced AKI, miRNA microarray assays were performed in order to detect miRNAs. Kidney-enriched miRNAs deriving from rat plasma were used as biomarkers for contrast-induced AKI. Results obtained from this animal model were further validated in patients with contrast-induced AKI [27]. Among the 51 miRNAs expressed in the rat renal tissue, only miR-30a, miR-30c, and miR-30e showed a >2-fold increases in contrast-induced AKI patients when compared with controls, with AUCs (area under the curve) of 0.954, 0.888, and 0.835, respectively [27]. This study has some limitations. First, although the recommended assessment of Scr at follow-up is 48–72 h for contrast-induced AKI, data obtained from the present study come from a follow up of 48 h after the administration of contrast medium. Moreover, patients with severe renal dysfunction (eGFR < 30 mL/min/1.73 m^2^) were excluded, so further studies are needed in this population of patients. Nevertheless, these miRNAs might be considered good diagnostic biomarkers in the setting of contrast-induced AKI. 

Similarly, Shi-Qun Sun et al. demonstrated a >1.5-fold increase in plasma levels of miRNA-188, miRNA-30a, and miRNA-30e in rats with contrast-induced AKI [28]. The peak was recorded around 4 h after contrast medium exposure and it was relatively renal-specific. The plasma levels of these miRNAs were then analyzed in 71 patients developing AKI after coronary angiography or percutaneous coronary intervention and in 71 matched controls. MiRNAs’ plasma levels were significantly higher in the AKI group as compared to the control group [28]. These data support the results of the previous study in the possible role of these miRNAs in the early diagnosis of contrast-induced AKI. Anyway, this study has several limitations. First, the peak values of these circulating miRNAs occurred 4 h after contrast medium exposure, then dropping quickly. This narrow time-window for miRNA detection might limit their clinical utility as biomarkers for disease monitoring. Second, baseline hydration status play a pivotal role in the development of contrast-induced AKI. However, parameters regarding the hydration status, such as left ventricular end-diastolic pressure, were not reported. Finally, this is a single-center, small sample-size study without prognosis information.

In patients developing AKI after cardiac surgery, urine miR-30c-5p and miR-192-5p levels increase 2 h after the procedure, thus suggesting their possible role in the prediction of renal damage after the procedure [29]. Urine samples were collected in 71 patients undergoing cardiac surgery at the beginning of operation (0 h) as well as 2 h after the procedure. The quantitative validation of microRNA showed a significant elevation in the urinary expression of miR-30c-5p and miR-192-5p 2 h after the operation, and earlier than kidney injury molecule-1 [29]. Importantly, in this study the candidate miRNAs were screened in a controlled animal model of ischemia-reperfusion-induced kidney injury rather than in patients with AKI in order to ensure the purity of the cause of renal damage, and to avoid possible effects of comorbidities on the spectrum of urine miRNAs [29].

In addition, Du et al. highlighted the association between urine and plasma miR-21 levels and the development and progression of AKI in patients undergoing cardiac surgery [30]. In this study, urine and plasma levels of miR-21 were obtained by quantitative real-time PCR in 120 adult patients undergoing cardiac surgery, who were divided in non-AKI controls, patients with progressive AKI, and with non-progressive AKI. An increase in both urine and plasma levels of miR-21 in patients with AKI was observed in patients with the progression of the disease. Indeed, the AUCs for urine and plasma levels of miR-21 associated with established AKI were 0.68 (95%CI: 0.59–0.78) and 0.80 (95%CI: 0.73–0.88), respectively. Urinary and plasma miR-21 levels were also good predictors of the need for postoperative renal replacement therapy, 30-day in-hospital mortality and prolonged stay in hospital or in intensive care unit [30]. One limitation of this small single center study is the absence of data on anuric and oliguric patients or those with a baseline eGFR < 30 mL/min/1.73 m^2^.

MiRNA has been demonstrated to be involved in the pathogenesis of ischemia/reperfusion (IR) induced kidney damage. In particular, miR-330-5p is stimulated by long non-coding RNA 122049 to increase the expression of ETS transcription factor ELK1 (ELK1), thus reducing renal cell apoptosis in the setting of ischemic AKI [31]. The miR-330-5p/ELK1 axis may represent a potential target in the treatment of ischemic renal damage [32].

Moreover, the upregulation of miR-21 in renal ischemia/reperfusion injury (IRI) may be considered as an anti-apoptotic factor in delayed ischemic preconditioning in mice [31]. Indeed, knockdown of miR-21 is associated to a significant upregulation of programmed cell death protein 4, with consequent increased tubular cell apoptosis [31]. In the setting of IRI, miR-21 may represent a novel pharmacological potential target.

An animal study on p53/miR-17-5p/DR6 pathway in renal IRI demonstrated an upregulation of this molecule in hypoxic renal tubular cells. In in-vitro studies, miR-17-5p inhibited death receptor 6 (DR6) and diminished apoptosis during hypoxia. In-vivo mimickers of miR-17-5p suppressed DR6 activity and protected against IRI. Role of miR-17-5p is dependent of p53 during an IRI event. A 48-h study revealed induction was greatest at 24 h post ischemia treatment [33].

Kidney transplant-associated AKI represents a complex syndrome which may be triggered by ischemic, immunologic, nephrotoxic and infectious insults [34,35,36,37]. In the setting of acute rejection, urinary levels of miR-155 have been investigated in the study performed by Wilflingseder et al. [34]. The authors identified distinct deregulated miRNAs in human renal allograft biopsies from patients undergoing acute cellular rejection, antibody-mediated rejection (ABMR), and delayed graft function (DGF). MiR-155 up-regulation was unique to T cell-mediated rejection, while miR-125 was downregulated both in DGF and ABMR [34]. A limitation of this study is in the interpretation of the given kidney biopsy miRNA profiles, specifically the incomplete human miRNA list and their experimentally validated target lists. Anyway, these findings support the potential diagnostic and therapeutic use of these biomarkers in the setting of kidney transplant-associated AKI (Table 2).

Additionally, Lorenzen et al. demonstrated an inverse correlation between urinary miR-210 levels and the severity of acute kidney rejection, as well as a normalization in its concentrations after successful treatment [38]. On the contrary, lower levels of miR-210 were associated with long-term decline in graft function [38]. A limitation of this study is the lack of molecular insights into the underlying mechanisms causing a dysregulation of urinary miRNAs in patients with acute rejection. Nevertheless, miR-210 may represent a potential diagnostic biomarker of acute kidney rejection, and a predictive factor of therapy response.

**Table 1:** The role of miRNAs in the setting of AKI. AKI (acute kidney injury); RUNX1 (runt-related transcription factor 1); SNHG5 (small nuclear RNA host gene 5); TLR4/NF-ĸb (toll-like receptor 4/nuclear factor-ĸB); I/R (ischemia/reperfusion); DR6 (death receptor 6).

miRNA	Effect	AKI Model	Origin of Sample	Reference
miR-21-5p	Decrease in serum inflammation, apoptosis and oxidative stress through RUNX1	Sepsis-induced AKI	Serum and kidney tissue	Zhang et al., 2021 [24]
miR-374a-3p	Decrease in apoptosis and cytokines release through a down-regulation of SNHG5 through the axis TLR4/NF-ĸB	Sepsis-induced AKI	Serum	Wang et al., 2021 [25]
miR-29a and -10a-5p	Good predictors for 28-day survival	Sepsis-induced AKI	Serum	Huo et al., 2017 [26]
miR-30a, -30c, and -30e	>2-fold increase in contrast-induced AKI patients when compared with controls	Contrast-induced AKI	Plasma and kidney tissue	Gutiérrez-Escolano et al., 2015 [27]
miR-188, -30 and -30e	>1.5-fold increase in contrast-induced AKI patients when compared with controls	Contrast-induced AKI	Plasma and kidney tissue	Sun et al., 2016 [28]
miR-30c-5p and -192-5p	Significant increase in urine levels 2 h after cardiac surgery	Post-operative AKI	Urine	Zou et al., 2017 [29]
miR-21	An increase in urine and plasma levels is associated with the development and progression of AKI and predicts the need for postoperative renal replacement therapy, 30-day in-hospital mortality and prolonged stay in hospital or in intensive care unit	Post-operative AKI	Urine and plasma	Du et al., 2013 [30]
miR-330-5p	Reduction of renal cell apoptosis in the setting of ischemic AKI, stimulated by long non-coding RNA 122049	I/R injury	Serum and kidney tissue	Xiao et al., 2022 [32]
miR-21	Antiapoptotic effect in delayed ischemic preconditioning in mice	I/R injury	Serum and kidney tissue	Xu et al., 2012 [31]
miR-17-5p	Antiapoptotic effect during hypoxia through the inhibition of the expression of DR6	I/R injury	Kidney tissue	Hao et al., 2018 [33]
miR-874-3p	Attenuation of renal tubular epithelial cell injury and enhancement of repair mechanisms after kidney injury	Cisplatin induced AKI	Serum and kidney tissue	Yu et al., 2023 [39]
miR -24, -126, -494 and -687	Regulation of inflammation, cell cycle and programmed cell death in the repair stages of AKI	AKI progression	Serum	Ren et al., 2018 [40]
miR-21	Its upregulation promotes renal fibrosis through the suppression of peroxisome proliferator-activator receptor alpha and phosphatase and tensin homolog	AKI progression	Serum	Lv et al., 2018 [41] McClelland et al., 2015 [42]
miR-29a, -29b and -29c	Antifibrotic effect	Renal fibrosis in diabetic nephropathy	Kidney tissue	Wang et al., 2012 [43]

MiRNA-21 is the most frequently investigated miRNA as diagnostic biomarker and as therapeutic target, impacting apoptosis, inflammation and renal fibrosis. MiRNA-30 family has also been deeply studiedas potential biomarker in contrast-induced and post-operative AKI. In the setting of AKI progression towards CKD, miRNA-29 family has been demonstrated to play a pivotal role, thus representing a potential pharmacological target.

**Table 2:** The role of miRNA in the different types of AKI.

AKI Etiology	miRNA	Role in Clinical Practice
Sepsis	miR-21-5p, miR-374a-3p	Therapeutic targets
	miR-29-a, miR-10a-5p	Mortality predictors
Contrast-induced AKI	miR-30a, miR-30c miR-30e, miRNA-188	Diagnostic biomarkers
	miR-30c-5p, miR-192-5p	Diagnostic biomarkers
Cardiac surgery	miR-21	Predictor of AKI progression, the need of RRT, mortality and prolonged stay
Ischemia/Reperfusion injury	miR-330-5p miR-21, miR-17-5p	Therapeutic targets
Kidney transplant-associated AKI	miR-155, miR-125	Diagnostic biomarkers
	miR-210	Diagnostic biomarker and therapeutic target

### 4.2. MiRNAs Involved in Fibrosis Progression and CKD

AKI represents a risk factor for the progression of kidney disease, and, in this context, cellular proliferation is fundamental to restore tubular integrity. MiR-874-3p has been shown to mitigate renal tubular epithelial cells damage induced by the administration of cisplatin in an animal model [39]. In previous studies, the role of human umbilical cord mesenchymal stem cells in kidney injury has been investigated. In a rat model, epithelial cell injury was induced by cisplatin. Human umbilical cord mesenchymal stem cell exosomes inhibited necroptosis after renal tubular epithelial cell injury and promoted cellular repair thanks to the maintenance of mitochondrial functional homeostasis, through the activity of miR-874-3p [39]. The induction of miR-874-3p may represent a pharmacological target in the prevention of acute kidney injury progression towards chronic kidney disease.

Moreover, miR-24, miR-126, miR-494, and miR-687, may regulate inflammation, cell cycle, and programmed cell death in the different repair stages of AKI through the binding to the 3′-untranslated region of their target genes. This highlights their therapeutic potential in AKI [40].

MiRNAs seem to play a pivotal role also in renal fibrosis which characterizes the progression of all kidney disease. Animal studies have demonstrated an up-regulation of mi-R21 in diabetic kidney disease while its deficiency seems to be protective against renal fibrosis [41]. Specifically, the profibrotic effects of mi-R21 result from the suppression of peroxisome proliferator-activator receptor alpha (PPAR)-α and phosphatase and tensin homolog and small mothers against decapentaplegic homolog 7 [42]. PPAR-α is both one of the most potent transcription factors regulating fatty acid oxidation and a validated target of miR-21. In normal kidneys, high PPAR-α levels are generally found in interstitial cells, whereas after acute renal injury their expression decreases secondarily to miR-21 targeting. The upregulation of PPAR-α expression in transgenic mice subjected to unilateral ureteric obstruction or ischemic injury reduces renal interstitial fibrosis. These data support the importance of fatty acid oxidation in the prevention of renal disease progression and the fundamental role of miR-21 silencing of the PPAR-α axis [41].

On the other hand, miR-29a, miR-29b and miR-29c have been demonstrated to be antifibrotic factors in diabetic nephropathy, membranous nephropathy, focal segmental glomerulosclerosis and IgA nephropathy [43]. The expression of miR-29 has been demonstrated to reduce the production of collagens I and IV at both the mRNA and protein levels by targeting the 3′untranslated region of these genes. Pharmacologic modulation of these miRNAs may have therapeutic potential in the prevention of progressive renal fibrosis.

## 5. Limitations and Clinical Challenges

We acknowledge certain weakness that warrant caution when interpreting the data reported. Concerns about miRNA isolation and measurement methods need to be mentioned. The wide variety of biological sources of miRNAs, the interindividual variability in their expression, and the lack of standardization of collection can all influence data reproducibility. Indeed, data normalization, a process used in RT-qPCR to minimize technical variability between samples, was not standardized across the studies, thus impacting miRNA reporting. Also costs and feasibility of adoption should be considered in clinical practice. Another issue which should be raised regards the involvement of miRNAs in other disease, such as cancer, cardiovascular and autoimmune disease and liver fibrosis. This could affect the reliability of miRNAs as biomarkers of AKI.

## 6. Extracellular Vesicles and Circulating miRNAs

As notable discovery, microRNAs have been found in the extracellular space and in biological fluids, such as serum, plasma, urine, saliva, and cerebrospinal fluid. They usually keep a relatively stable condition in spite of the existence of RNAse which are enzymes that typically degrade RNA [44]. The inclusion in extracellular vesicles (EVs), such as microvesicles, exosomes and apoptotic bodies, as well as the formation of protein-microRNA complexes, represent possible mechanisms against RNAse degradation [45].

**Figure 2:**
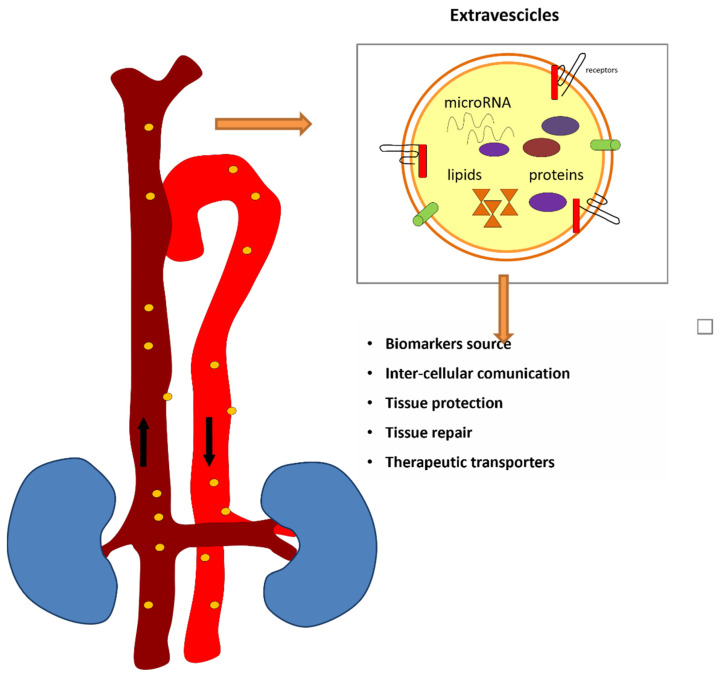
MiRNA transport via EV.

EVs are efficient tools for miRNA stabilization, transport, and delivery, playing an essential role in intercellular communication and gene regulation. Indeed, EVs protect miRNAs from degradation by encapsulating them within a lipid bilayer. This shielded environment significantly enhances the stability of miRNAs in the bloodstream. Furthermore, EVs act as vehicles for the transport of miRNAs to target cells. By packaging miRNAs into EVs, they can travel through the bloodstream and deliver their cargo to distant cells, maintaining their functional integrity. The vesicles facilitate the transfer of miRNAs between cells without the risk of degradation by extracellular enzymes. Moreover, EVs contain surface proteins and ligands that can interact with receptors on the target cell membranes. This interaction can facilitate the uptake of miRNA-loaded EVs by recipient cells, thus preserving the biological activity of miRNAs during circulation [46,47] (Figure 2).

Extracellular vesicles are cell-derived vesicles walled in a lipid bilayer, with a diameter ranging from 30 nm to 2000 nm depending on the origin [48,49]. They contain widespread proteins linked to biogenesis and trafficking, as well as precise cellular and tissue factors, for example specific proteins and DNA and RNA. Thus, the learning of the content of EVs in terms of proteome and nucleic acid may offer information on the cell or tissue of origin and, importantly, on their physiological condition. This information is essential for understanding the biological processes that are taking place in the body and can offer a window into both normal cellular functions and pathophysiological changes. Exosomes and small non-coding RNAs might participate in the pathophysiology of kidney disease, mediating intercellular communication and signaling mechanisms in the target cells, transferring genetic materials and proteins [44,45,46,47,48,49]. Through this complex communication network, exosomes and their cargo contribute significantly to both renal health and disease progression, influencing various biological processes that drive the onset and progression of kidney disorders.

Recent studies have suggested that EVs play a crucial role in the pathophysiology of AKI. These extracellular particles carry important molecular signals and influence the progression of AKI through several mechanisms, such as inflammation, oxidative stress, apoptosis and repair mechanisms [50,51,52,53,54,55,56]. Generally, EVs can influence the inflammatory response in AKI by transporting pro-inflammatory cytokines and immune signals and interacting with immune cells, potentially exacerbating or mitigating kidney damage [50,51,52,53]. In a study conducted at the Royal Brisbane and Women’s Hospital in Australia, researchers observed that hypoxic tubular epithelial cell (TEC)-derived EVs, through the transfer of specific miRNAs and other bioactive molecules, intensify oxidative stress and tissue damage in the kidney [56]. This process potentially drives the transition from AKI to CKD. Moreover, Jeon et al. reported that endothelial cells damaged by injured podocytes release EVs containing specific microRNAs, in particular miR-424 and miR-149. These may induce the p38 MARK signaling pathway activation, leading to apoptosis in renal tubular epithelial cells [57].

Interestingly, beyond their role in kidney injury, EVs have been implicated in the repair of damaged renal tissue. They can carry pro-regenerative molecules, such as growth factors and microRNAs, that promote tissue regeneration and repair after injury, potentially aiding in recovery from AKI [58]. Given their critical role in inflammation, apoptosis, and repair, EVs present significant potential in the diagnosis and therapy of AKI. They could serve as biomarkers for early detection of AKI, even before noticeable symptoms occur. In addition to their diagnostic potential, the therapeutic applications of EVs are being actively explored. Research is investigating their use in targeted drug delivery systems, where EVs can be engineered to carry therapeutic agents to specific sites within the kidneys [59,60,61,62].

Microvesicles (MVs) transfer bioactive genetic information from one cell to another by means of mRNA, miRNA and certain transcription factors. Indeed, MVs serve as vehicles for the transportation of genetic material, which can influence the function and behavior of recipient cells. The transfer of genetic information through MVs has a profound impact on various biological processes, including immune modulation, inflammation, cancer progression, tissue repair, and organ homeostasis. This process is undertaken by acting as a signaling complex or by transferring surface receptor determinants. This led to horizontal transfer of genetic information causing epigenetic changes. Epigenetics refers to heritable changes in gene expression or cellular phenotype that occur without alterations to the underlying DNA sequence. MVs are able to carry specific molecular signals that can influence epigenetic mechanisms, such as DNA methylation, histone modifications, and non-coding RNA regulation. MVs from the endothelium can transfer mRNA and modulate the behavior of the target cell. This property came into light by the ability of RNAse to inhibit MV associated biological effect [48].

Certain studies have shown that MVs originating in stem cells not only carry mRNA but also carry miRNA which have the ability of gene silencing and modulating protein translation [63]. Urinary exosomes are now serving as biomarkers in renal pathologies. Urinary exosomal fetuin-A can indicate cisplatin induced nephrotoxicity and AKI patients in ICU. A reduction in aquaporin-1 in urinary exosomes in rats has shown to indicate IR injury in kidneys [48].

## 7. Limitations and Clinical Challenges

The practical application of exosome-based approaches in clinical practice faces significant challenges, particularly in the isolation and quantification of these vesicles. The effectiveness and reproducibility of using exosomes in diagnostics or therapeutics depend heavily on overcoming these hurdles. Several methods exist for isolating exosomes from biological samples: ultracentrifugation, Density Gradient Centrifugation, Immunoaffinity-based Isolation, Size-Exclusion Chromatography, Microfluidics, Precipitation-Based Methods, Filtration-Based Methods. Each exosome isolation method has its strengths and weaknesses and its own advantages and limitations, making the choice of technique dependent on the specific requirements of the study, such as purity, throughput, and available resources. For clinical applications, methods that offer high specificity, reproducibility, and scalability, such as microfluidics and immunoaffinity-based methods, are being increasingly explored. However, traditional methods like ultracentrifugation remain a reliable and widely used choice in both research and clinical settings. The choice of isolation technique also needs to consider the intended downstream applications (e.g., proteomic, genomic, or functional analyses) to ensure the most appropriate method is selected.

In the same way, several methods, such as Nanoparticle Tracking Analysis, Flow Cytometry, Western Blotting, Quantitative PCR (qPCR), RNA Sequencing (RNA-Seq), exist for quantification of EVs. Standardization issues and method selection can impact the accuracy, reproducibility, and clinical applicability of exosome-derived miRNA quantification. However, standardization of the isolation, quantification, and normalization processes is critical for ensuring reproducibility, comparability, and clinical applicability. While methods like RT-qPCR are commonly used due to their sensitivity, RNA sequencing offers a more comprehensive approach, albeit with greater complexity and cost. Addressing the standardization issues in miRNA quantification will enhance the clinical and research utility of exosome-derived miRNAs as biomarkers and therapeutic targets [64,65,66,67].

A key barrier to the widespread clinical implementation of exosome-based diagnostics is the lack of standardized protocols for both isolation and quantification. Without standardized guidelines, the reproducibility and reliability of exosome-derived biomarker assays are compromised, making it challenging to translate research findings into clinical practice.

## 8. Conclusions

Despite the big amount of literature about miRNAs, there is still need for further studies evaluating their role in both diagnosis and treatment of AKI. The study of miRNAs will help us to assess the feasibility and the usefulness of using a non-invasive method to predict a clinical condition whose morbidity and mortality are still high. The discovery of specific miRNAs in serum, urine, and plasma, has opened up promising avenues for the development of non-invasive diagnostic tools.

However, much of the current research on miRNAs has focused on single individual biomarker, and there is still a gap in understanding the comprehensive molecular miRNA signature that can accurately reflect the clinical status of AKI across different patient populations. The use of miRNA panel, based on multiple miRNAs that act together, needs to be more fully explored to increase the accuracy of prediction. This approach could overcome the limitations of individual markers by incorporating a broader array of molecular signals that reflect multiple pathways involved in AKI, including inflammation, oxidative stress, vascular injury, and tubular damage.

Future research should focus on improving study design in human AKI and optimizing methods for EVs and miRNA isolation, detection and quantification. Overall, miRNAs could represent potential biomarkers for the early diagnosis of AKI and new therapeutical targets in this clinical setting.

## Abbreviations

The following abbreviations are used in this manuscript:

AKI	Acute kidney injury
CKD	Chronic kidney disease
IR	Ischemia–reperfusion
IRI	Ischemia–reperfusion injury
miRNA	MicroRNA
Evs	Extracellular vesicles
MVs	Microvesicles
piRNA	Piwi-interacting RNA
rasi-RNA	Repeat-associated small interfering RNA
si-RNA	Small interfering RNA
sco-RNA	Small cell osteosarcoma RNA
sn-RNA	Small nuclear RNA
sno-RNA	Small nucleolar RNA
ti-RNA	Transcription initiation RNA
